# Effect of sleeve gastrectomy, Roux-en-Y gastric bypass, and ileal transposition on myocardial ischaemia–reperfusion injury in non-obese non-diabetic rats

**DOI:** 10.1038/s41598-021-03283-y

**Published:** 2021-12-13

**Authors:** Oleg Kornyushin, Dmitry Sonin, Alexander Polozov, Vitaly Masley, Nika Bulavinova, Maria Chervyak, Maria Istomina, Daria Mukhametdinova, Alexander Neimark, Yuri Cheburkin, Yana Toropova, Kira Derkach, Alexander Shpakov, Michael Galagudza, Evgenyi Shlyakhto

**Affiliations:** 1 Institute of Experimental Medicine, Almazov National Medical Research Centre, Saint Petersburg, Russian Federation; 2 Laboratory of Physiology Nutrition, Pavlov Institute of Physiology RAS, Saint Petersburg, Russian Federation; 3 Pavlov First Saint Petersburg State Medical University, Saint Petersburg, Russian Federation; 4 Laboratory of Molecular Endocrinology and Neurochemistry, Sechenov Institute of Evolutionary Physiology and Biochemistry, Saint Petersburg, Russian Federation

**Keywords:** Cardiovascular diseases, Cardiovascular diseases, Gastrointestinal models

## Abstract

Bariatric surgery (BS) improves outcomes in patients with myocardial infarction (MI). Here we tested the hypothesis that BS-mediated reduction in fatal MI could be attributed to its infarct-limiting effect. Wistar rats were randomized into five groups: control (CON), sham (SHAM), Roux-en-Y gastric bypass (RYGB), sleeve gastrectomy (SG), and ileotransposition (IT). Ten weeks later, animals were subjected to 30-min myocardial ischemia plus 120-min reperfusion. Infarct size (IS) and no-reflow area were determined histochemically. Fasting plasma levels of glucagon-like peptide-1 (GLP-1), leptin, ghrelin, and insulin were measured using ELISA. Compared with SHAM, RYGB and SG reduced IS by 22% (p = 0.011) and 10% (p = 0.027), and no-reflow by 38% (p = 0.01) and 32% (p = 0.004), respectively. IT failed to reduce IS and no-reflow. GLP-1 level was increased in the SG and RYGB groups compared with CON. In both the SG and RYGB, leptin level was decreased compared with CON and SHAM. In the SG group, ghrelin level was lower than that in the CON and SHAM. Insulin levels were not different between groups. In conclusion, RYGB and SG increased myocardial tolerance to ischemia–reperfusion injury of non-obese, non-diabetic rats, and their infarct-limiting effect is associated with decreased leptin and ghrelin levels and increased GLP-1 level.

## Introduction

Although bariatric surgery (BS) was initially intended as a procedure for weight reduction, it has now evolved into a metabolic surgery that allows achieving not only a 62% reduction in body weight but also a 76.8% and 61.7% remission of type 2 diabetes mellitus (T2DM) and arterial hypertension, respectively^1,2^. An important indicator of the success of BS for obesity treatment is a decrease in the rates of cardiovascular events and mortality^3,4^. The decrease in cardiovascular disease incidence after BS is confirmed by data showing decreased occurrences of myocardial infarction (MI) and chronic heart failure, as well as reduced number of patients needing emergency care for CHF, coronary artery disease, and hypertension^5,6^.

The metabolic effects of BS have been studied since the 1970s^7^. The data on the effects of BS on the severity of dyslipidaemia and on the course of hypertension (correction, remission, or amelioration) have suggested secondary (or mediated) mechanisms of the metabolic effects of BS on the cardiovascular system^1^. Initially, the metabolic effects of BS were explained as resulting from the associated food intake restriction or malabsorption^8^. However, recent data on the metabolic effects of gastrointestinal hormones and adipokines (including their cardioprotective effects) have suggested that BS may have direct myocardial effects^9^.

The metabolic effects of BS are known to result not only from a decrease in excessive body weight but also from the impact of the procedure on the levels of gastrointestinal hormones^10,11^. Currently, the most studied incretin hormone is glucagon-like peptide-1 (GLP-1), which has pronounced trophic and protective effects on the pancreatic β-cells, myocardium, brain, and endothelial system^12–14^. The hormones of the ghrelin family (ghrelin and obestatin) also have cardioprotective effects^15–17^. The Swedish Obesity Study reported a significant reduction in fatal MI after BS, which suggests an infarct-limiting effect^3^. In addition, BS has been shown to significantly contribute to the balance of gastrointestinal hormones that have potential cardioprotective effects^18^. In our previous study, we have shown that infarct size (IS) was smaller in the diabetic rats subjected to different types of BS; however, diabetes mellitus itself induced the infarct-limiting effect known as metabolic preconditioning, which complicated the interpretation of the results^19^.

After most types of BS, plasma GLP-1 level increases, and this effect is most pronounced after Roux-en-Y gastric bypass (RYGB) and is moderate after sleeve gastrectomy (SG). In contrast, the ghrelin level tends to decrease. After the removal of the ghrelin-producing zone, a more significant decrease in ghrelin level occurs after SG. Meanwhile, the existing data about the effect of RYGB on ghrelin levels are contradictory^18^. The purpose of the current study was to assess the myocardial tolerance of non-diabetic and non-obese rats to ischaemia–reperfusion injury after the most commonly performed BS procedures, including RYGB, SG, and ileal transposition (IT), which, along with their cardioprotective effect, can influence the balance and functional activity of the hormones of the gastrointestinal tract. Non-diabetic and non-obese animals were used to reduce the potential impact of humoral factors^20^ and metabolic preconditioning of hyperglycaemia^21,22^. IT was included in the analysis of this study because previous works have demonstrated pivotal role of GLP-1 elevation in the metabolic effects of this particular type of BS^23^.

## Results

### Body weight and food consumption

**Figure 1:**
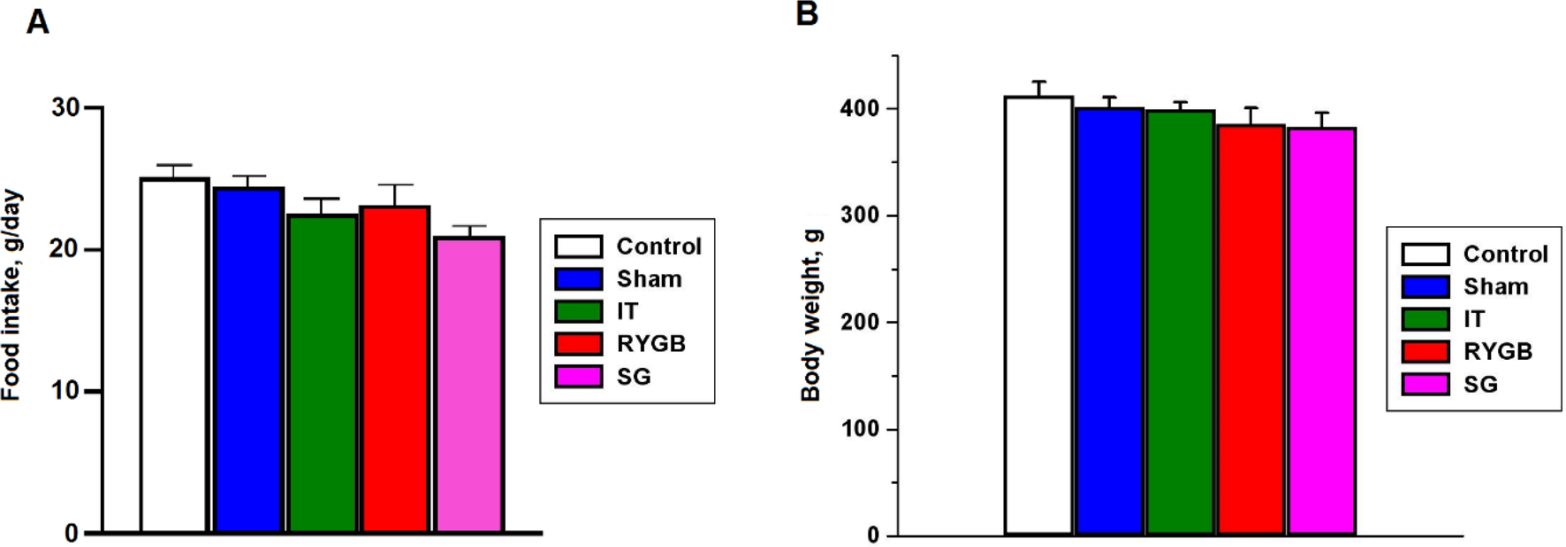
(**A**) Food consumption. (**B**) Effects of bariatric surgery on rat body weight. *CON* control, *SHAM* sham surgery, *IT* ileal transposition, *RYGB* Roux-en-Y gastric bypass, *SG* sleeve gastrectomy.

At 8 weeks after surgery, the food intake amount did not differ among the groups (Fig. 1A). The body weight at 10 weeks in all BS groups did not differ from that in the control (CON) and sham surgery (SHAM) groups (Fig. 1B).

### Effects of BS on plasma glucose, insulin, GLP-1, ghrelin, and leptin levels

**Figure 2:**
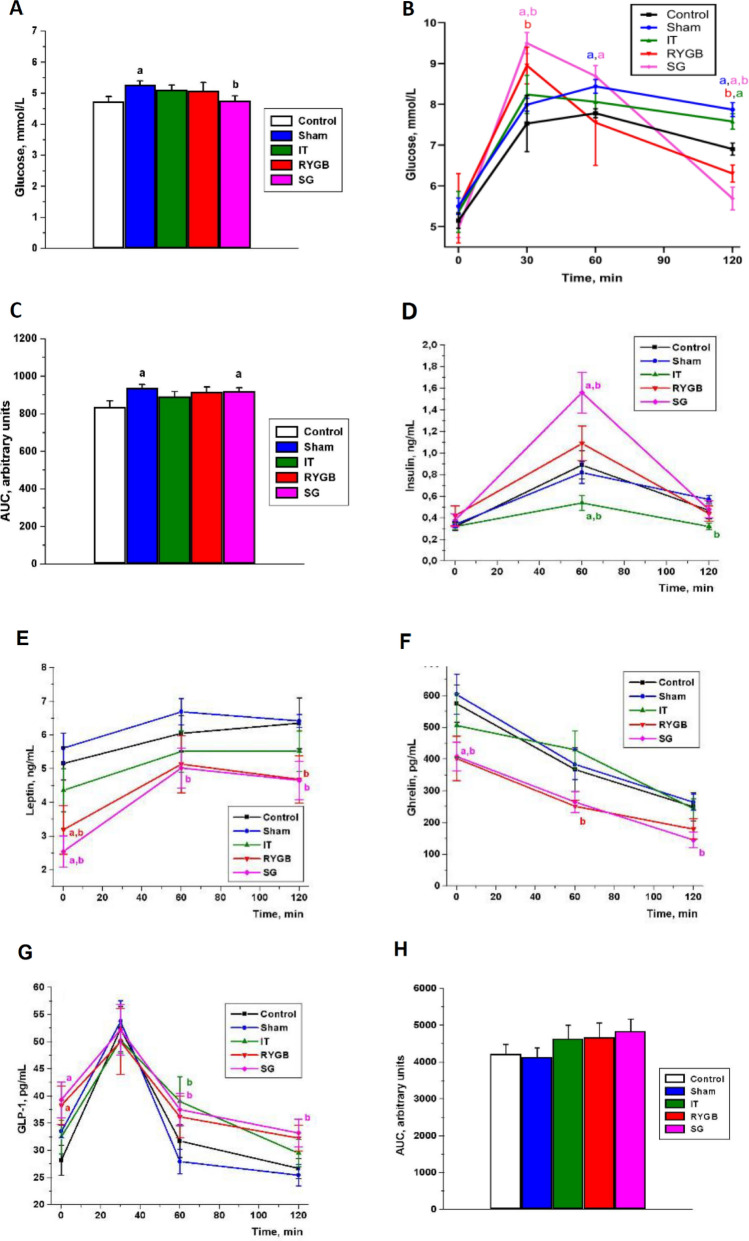
Levels of glucose and hormones in the oral glucose tolerance test. (**A**) Fasting plasma glucose levels. (**B**) Plasma glucose levels during oral glucose tolerance test. (**C**) Area under the curve of glucose levels. (**D**) Blood levels of insulin within 2 h after glucose loading. (**E**) Blood levels of leptin within 2 h after glucose loading. (**F**) Blood levels of ghrelin within 2 h after glucose loading. (**G**) Blood levels of GLP-1 within 2 h after glucose loading. (**H**) AUC of GLP-1 levels. (**a**) The difference between the control group and the other groups is significant at p < 0.05. (**b**) The difference between the sham surgery and bariatric surgery groups is significant at p < 0.05. Mean ± standard error of mean. *AUC* area under the curve, *CON* control, *SHAM* sham surgery, *IT* ileal transposition, *RYGB* Roux-en-Y gastric bypass, *SG* sleeve gastrectomy, *GLP-1* glucagon-like peptide-1.

The fasting glucose level was higher in the SHAM group than in the CON and SG groups (p < 0.05). In the SG group, the glucose level was lower than that in the SHAM group (p < 0.05, Fig. 2A) but did not significantly differ from that in the CON group (p > 0.05, Fig. 2A). In an oral glucose tolerance test (OGTT), the blood glucose level after 30 min was significantly higher in the SG and RYGB groups than in the CON and SHAM groups. Meanwhile, 90 min later, at 120 min of the OGTT, the glucose level significantly decreased in the SG group relative to the CON and SHAM groups and in the RYGB group relative to the SHAM group (for all, p < 0.05; Fig. 2B). In the SG and SHAM groups, the area under the curve (AUC) of the glucose level was significantly higher than that in the CON group (p < 0.05, Fig. 2C). In the SG group, the blood insulin level in response to glucose loading was higher than that in the CON and SHAM groups at 60 min after the start of the OGTT (p < 0.05, Fig. 2D). In contrast, in the IT group, the insulin level decreased at 60 and 120 min of the OGTT (p < 0.05, Fig. 2D). In the SG and RYGB groups, the fasting leptin level significantly decreased compared with the CON and SHAM groups (p < 0.05, Fig. 2E). In the SG group, the blood leptin level decreased during the entire OGTT in comparison with the SHAM group (p < 0.05, Fig. 2E). Meanwhile, in the RYGB group, a decrease in leptin level was observed only after 120 min (p < 0.05, Fig. 2E). In the SG group, the fasting plasma ghrelin level was lower than that in the CON and SHAM groups. At 120 min of the OGTT, the ghrelin level in the SG group was reduced compared with that in the SHAM group (p < 0.05, Fig. 2F). In the RYGB group, the ghrelin level was significantly lower than that in the SHAM group at 60 min of the OGTT (p < 0.05, Fig. 2F).

The fasting GLP-1 level was elevated in the SG and RYGB groups compared with that in the CON group (p < 0.05, Fig. 2G). After 60 min of the OGTT, the GLP-1 level was higher in the SG and IT groups than in the SHAM group; however, after 120 min, the level was higher only in the SG group (p < 0.05, Fig. 2G). Further, the AUC values of the GLP-1 level–time curves of the BS groups tended to be higher than those of the CON and SHAM groups (p > 0.05, Fig. 2H).

### Mortality and exclusions

**Figure 3:**
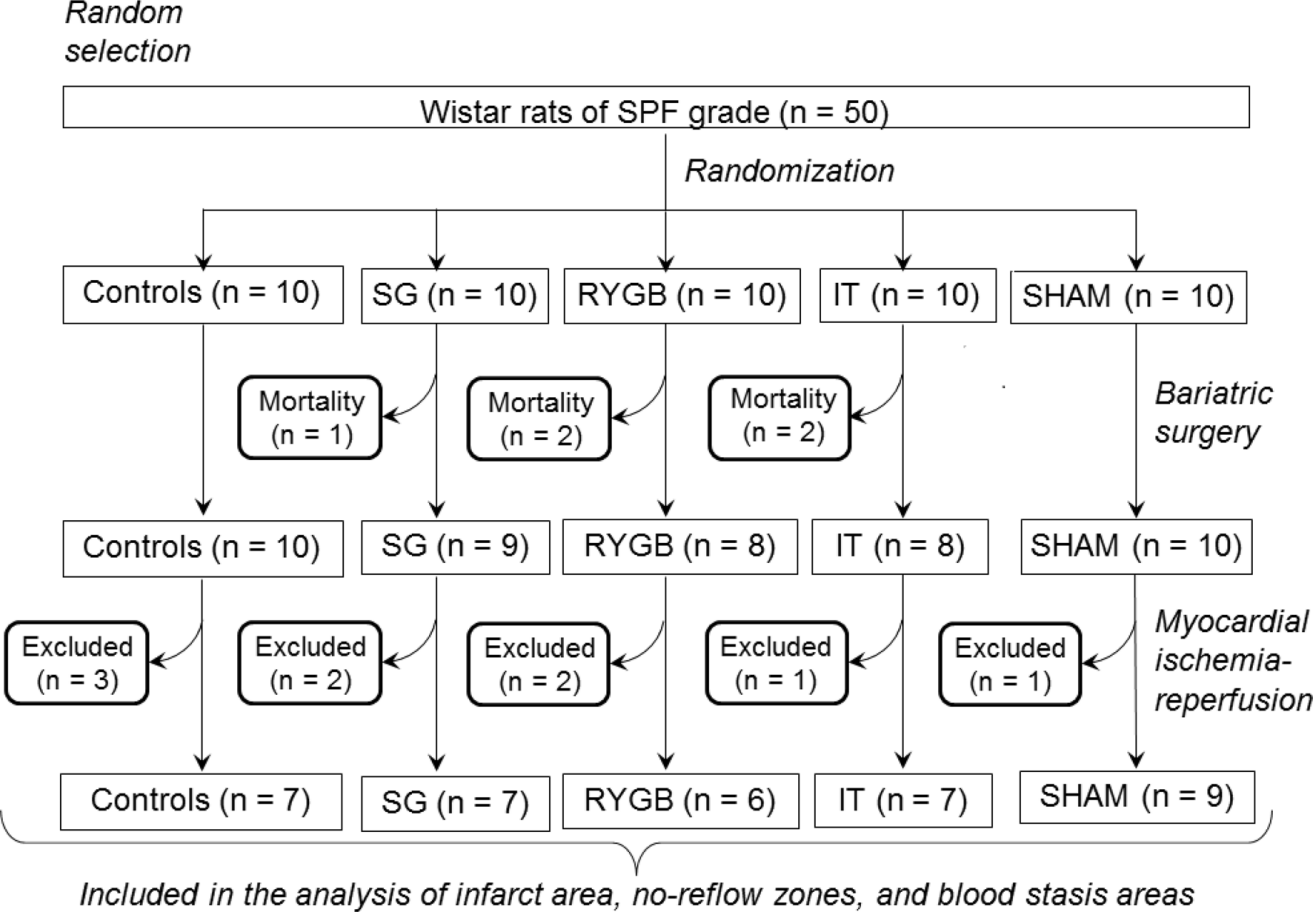
Flowchart showing the exact numbers of animals in each experimental group at different stages of the protocol, as well as the numbers of excluded/dead animals. *CON* control, *SHAM* sham surgery, *IT* ileal transposition, *RYGB* Roux-en-Y gastric bypass, *SG* sleeve gastrectomy.

A flowchart showing the exact numbers of animals in each experimental group at different stages of the protocol, as well as the numbers of excluded/dead animals, is presented in Fig. 3. In the case of SHAM and BS, the frequencies of postoperative mortality due to anastomotic leaks and/or strictures were 0/10, 2/10, 2/10, and 1/10 in the SHAM, IT, RYGB, and SG groups, respectively. At the stage of MI, the exclusion frequencies were 3/10, 1/10, 1/8, 2/8, and 2/9 in the CON, SHAM, IT, RYGB, and SG groups, respectively (Fig. 3). The main reasons for exclusion were persistent ventricular fibrillation, severe bleeding, hemodynamic instability, and area at risk (AR) < 15%. The number of animals included in the final analysis of the IS, no-reflow area, and blood stasis area was 7, 9, 7, 6, and 7 in the CON, SHAM, IT, RYGB, and SG groups, respectively (Fig. 3).

### Hemodynamic parameters

The baseline mean blood pressure levels did not differ among the groups (Table 1). During ischaemia and reperfusion of the myocardium, a progressive decrease in blood pressure was observed in all animals.

**Table 1:** Mean blood pressure values (mm Hg) in the experimental groups.

	Time, min	CON	SHAM	IT	RYGB	SG
Baseline	0	84 (83–90)	89 (74–96)	83 (75–90)	91 (83–96)	89 (74–96)
Ischemia	15	82 (72–88)	72 (70–81)	74 (68–79)	80 (70–89)	79 (71–88)
Reperfusion	40	76 (66–89)	74 (69–79)	75 (69–78)	69 (69–79)	76 (66–89)
	90	76 (72–84)	78 (68–81)	76 (71–89)	80 (72–87)	75 (71–78)
	180	75 (63–82)	76 (71–85)	68 (65–72)	74 (69–80)	77 (69–80)

Data are ‘median [25 quartile; 75 quartile]’.

*CON* control, *SHAM* sham surgery, *IT* ileal transposition, *RYGB* Roux-en-Y gastric bypass, *SG* sleeve gastrectomy.

### AR and IS

**Figure 4:**
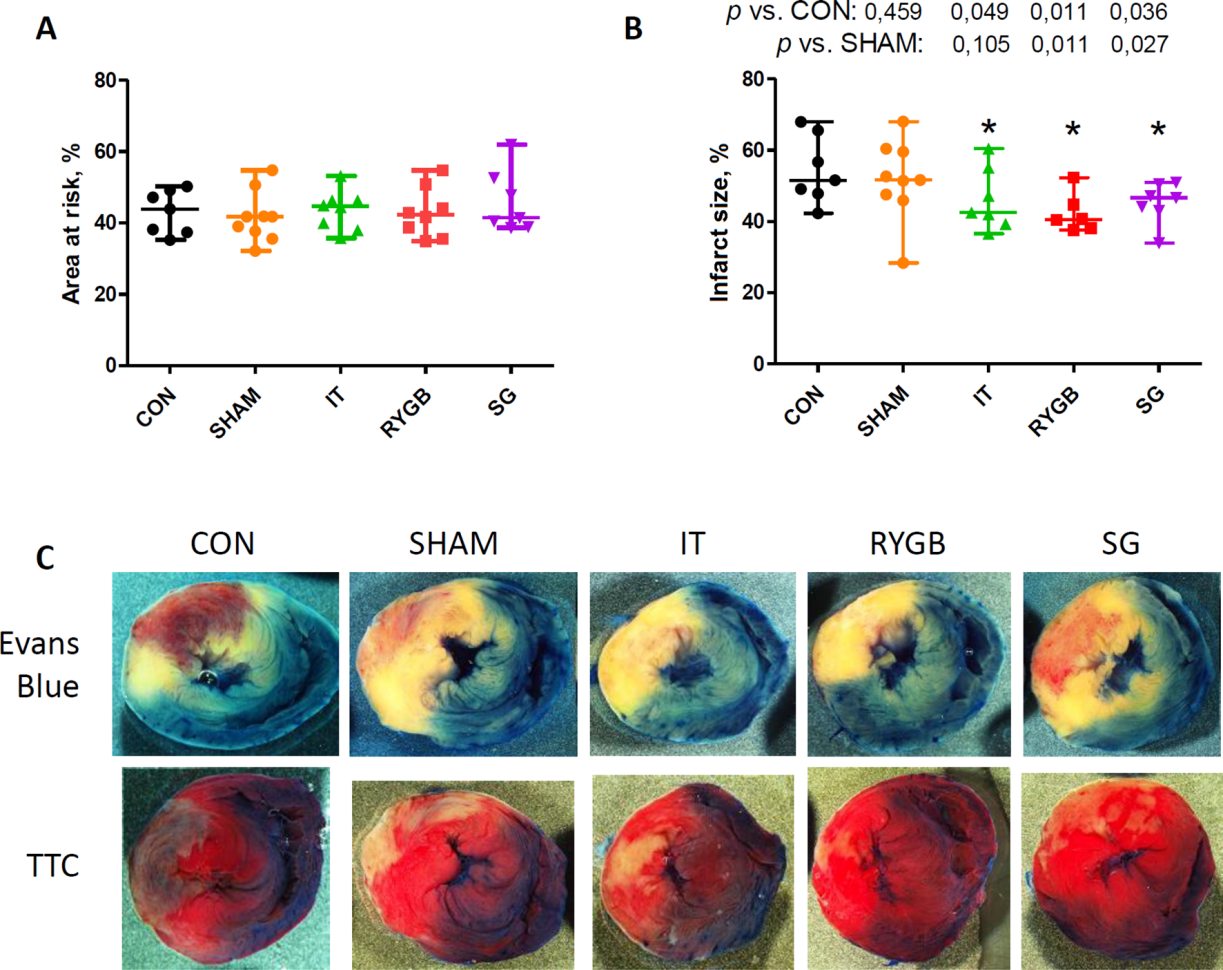
Myocardial area at risk and infarct size in the experimental groups. (**A**) Area at risk expressed as a percentage of the whole slice area. (**B**) Infarct size expressed as a percentage of the area at risk. (**C**) Representative images of heart slices stained with Evans blue and triphenyltetrazolium chloride. n = 7, 9, 7, 6, and 7 for the CON, SHAM, IT, RYGB, and SG groups, respectively. *p < 0.05 versus the CON group. *CON* control, *SHAM* sham surgery, *IT* ileal transposition, *RYGB* Roux-en-Y gastric bypass, *SG* sleeve gastrectomy, *TTC* triphenyltetrazolium chloride.

The size of the AR did not differ among the groups (Fig. 4). In all BS groups, the size of the IS was significantly smaller than that in the CON group (Fig. 4). In the SG and RYGB groups, the size of the IS was significantly smaller than that in the SHAM group (p = 0.011 and p = 0.027, respectively), whereas there was no significant difference in the size of the IS between the CON and SHAM groups.

### Sizes the no-reflow area and blood stasis area

**Figure 5:**
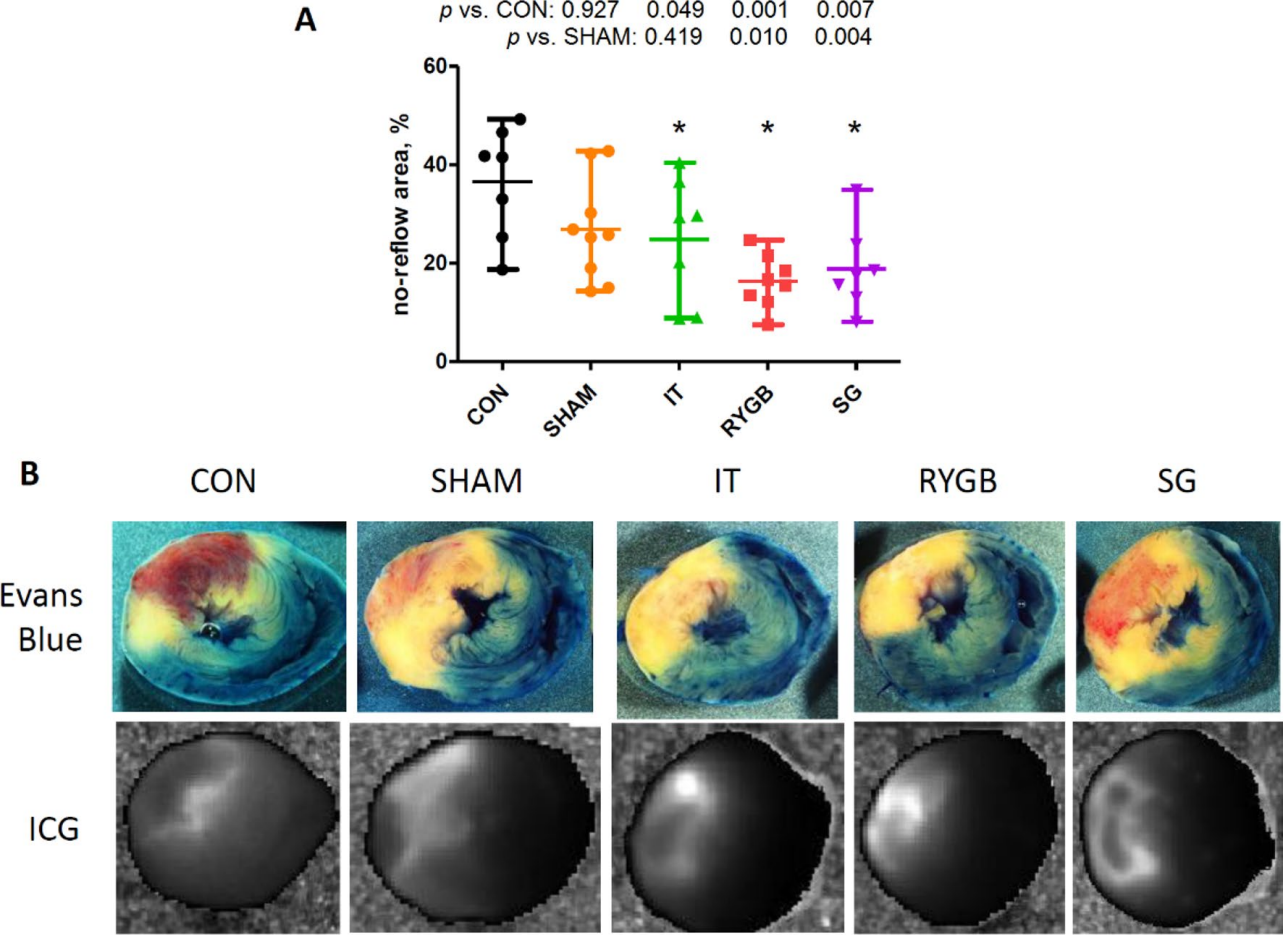
(**A**) Size of the no-reflow area expressed as a percentage of the area at risk. (**B**) Representative images of heart slices stained with Evans blue and indocyanine green. n = 7, 9, 7, 6, and 7 for the CON, SHAM, IT, RYGB and SG groups, respectively. *p < 0.05 versus the CON group. *CON* control, *SHAM* sham surgery, *IT* ileal transposition, *RYGB* Roux-en-Y gastric bypass, *SG* sleeve gastrectomy, *ICG* indocyanine green.

In all BS groups, the severity of the no-reflow phenomenon was less than that in the CON group (Fig. 5). In the SG and RYGB groups, the size of the no-reflow area was significantly smaller than that in the SHAM group (p = 0.010 and p = 0.004, respectively).

**Figure 6:**
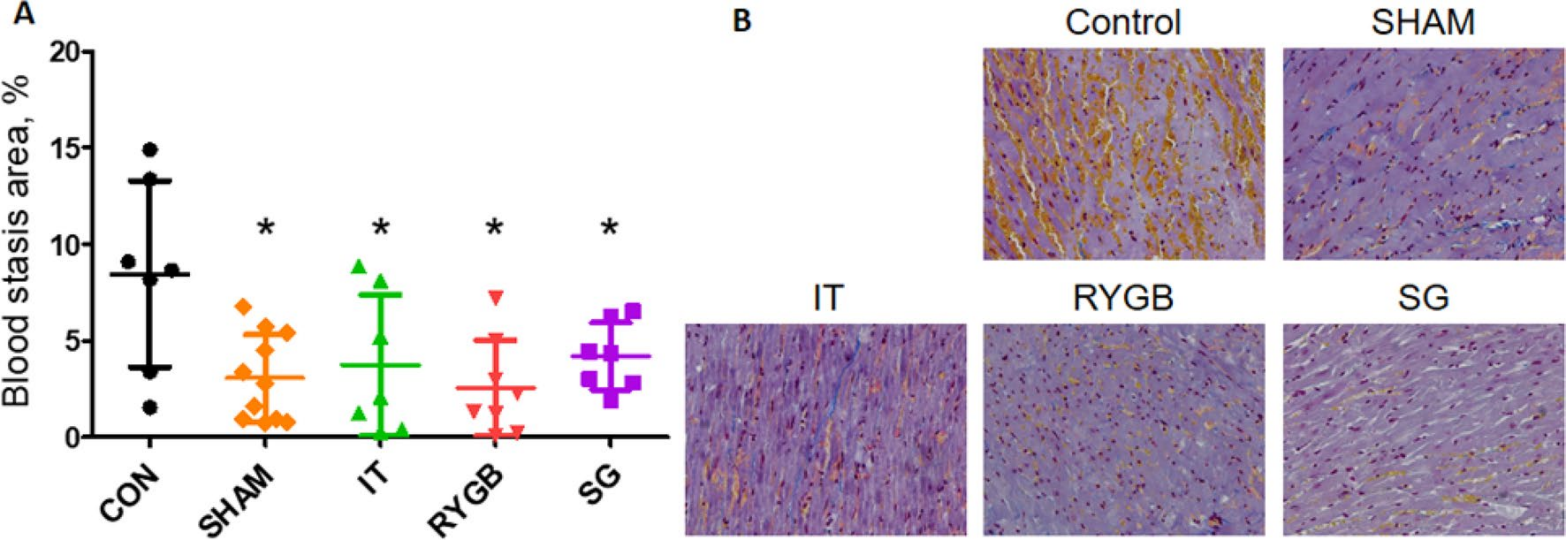
(**A**) Size of blood stasis areas expressed as a percentage of the total slice area after quantifying 10 visual fields in the area at risk. (**B**) Representative histological images demonstrating blood stasis. n = 7, 9, 7, 6, and 7 for the CON, SHAM, IT, RYGB and SG groups, respectively. *p < 0.05 versus the CON group. *CON* control, *SHAM* sham surgery, *IT* ileal transposition, *RYGB* Roux-en-Y gastric bypass, *SG* sleeve gastrectomy.

The blood stasis in the no-reflow area was significantly less in the experimental groups and in the SHAM group than in the CON group (Fig. 6).

## Discussion

BS decreases the risk of cardiovascular events by reducing the occurrence of metabolic disorders such as insulin resistance, impaired glucose tolerance, dysadipokinaemia, inflammation, and systemic lipotoxicity (including in the myocardium)^24^.

We hypothesized the existence of a link between changes in the profile of gastrointestinal hormones, leptin, and insulin after BS and a decrease in mortality from MI as a result of enhanced tolerance of the myocardium to ischaemia–reperfusion injury. In our study, an infarct-limiting effect and a decrease in the no-reflow phenomenon were observed in the SG and RYGB groups. We demonstrated, for the first time, that non-diabetic non-obese rats subjected to SG and RYGB before the induction of MI through coronary artery ligation showed reduced MI size after ischaemia and reperfusion (by 22% and 10% of the risk zone, respectively) and a decrease in the area of no-reflow (by 38% and 32%, respectively) compared with the SHAM group. These effects did not depend on changes in body weight. Table 2 summarizes the data on the effects of BS on myocardial tolerance to ischaemia–reperfusion injury and on the changes in the blood levels of several hormones with potential cardioprotective effects.

**Table 2:** Effect of various bariatric surgeries on the size of myocardial infarction and on the blood levels of hormones with potential cardioprotective effects in non-obese non-diabetic rats.

Effect	IT	RYGB	SG
	The difference between the SHAM and BS groups		
Sizes of zones IM	n.s	↓↓	↓↓
No-reflow	↔	↓↓	↓↓
GLP-1 fasting	↔	n.s	n.s
GLP-1 30 min	↔	↔	↔
Ghrelin fasting	↔	n.s	↓
Ghrelin 60 min	↔	↓	n.s
Leptin fasting	n.s	↓↓	↓↓
Leptin 60 min	n.s	n.s	↓
Insulin fasting	↔	↔	↔
Insulin 60 min	↓	↔	↑

n.s. = not statistically significant, ↑ = increased blood levels, ↓ = decreased blood levels, ↔  = unchanged blood levels, *GLP-1* glucagon-like peptide-1, *RYGB* Roux-en-Y gastric bypass, *IT* ileal transposition, *SG* sleeve gastrectomy.

Decreased plasma ghrelin and leptin and increased GLP-1 levels were observed in post-BS rats. The rats in the SG and RYGB groups showed an increased postprandial glucose level, but only for a short period as rapid normalization of the glucose level occurred 1 h after the OGTT. The increase in the glucose level at 30 min of the OGTT was accompanied by a significant increase in insulin secretion at 60 min, without subsequent hypoglycaemia after an hour of observation (at 120 min). All hormones analyzed in this study can affect the function of the cardiovascular system and can be cardioprotective to varying degrees. Below, we will compare our findings with the literature data on the effects of various BS procedures on the levels of these hormones. We will also discuss evidence of their possible contributions to the cardioprotective effects of SG and RYGB.

### Leptin

The decreased plasma level of leptin after BS restores leptin and insulin sensitivity and favorably affects the prognosis of patients with metabolic syndrome^25,26^. It should be noted that data on the effect of leptin on myocardial ischaemia–reperfusion injury remain controversial^27^. A number of studies reported that exogenous leptin decreased the IS when it was administered at the beginning of reperfusion of isolated mouse and rat hearts^28,29^. Smith et al. showed that the cardioprotective effect of leptin is associated with the activation of the Janus kinase/signal transducer and activator of transcription (JAK/STAT) signalling pathway, leading to a decrease in mitochondrial pore permeability. Inhibition of the JAK/STAT signalling pathway abolishes the leptin-induced cardioprotective effect^28^. There is also evidence that leptin-mediated cardioprotection involves activation of the reperfusion injury salvage kinase (RISK) pathway^30^. In 2010, Xu et al. showed that intraperitoneal administration of leptin 30 min before myocardial ischaemia–reperfusion in mice reduced reperfusion-induced inflammation and myocardial injury^31^.

Meanwhile, in 2018, Kain et al. demonstrated that postinfarction remodelling and myocardial dysfunction occurred due to mRNA overexpression of the leptin gene in cardiomyocytes^20^. The negative effect of leptin on the processes of postinfarction remodelling was confirmed by experiments in rats through the inhibition of cardiac leptin expression^27,32^. The authors showed that impairing leptin function in rats with acute MI led to reduced postinfarction remodelling. Chronically elevated leptin levels induced leptin resistance^33^. In contrast, a high-fat/cholesterol diet-induced increase in leptin level was associated with decreased tissue sensitivity to leptin^34^. In 2013, Nausheen et al. found that the blood levels of leptin in Sprague–Dawley rats were decreased by 57% after SG and by 59–72% after IT^35^.

Our data indirectly suggest that a decrease in the plasma leptin level in post-BS rats might be involved in the reduction of IS and the no-reflow phenomenon in the SG and RYGB groups. In the present study, a comparable decrease in leptin was observed in the SG and RYGB groups; notwithstanding, in the IT group, in which a significant decrease in the infarction size was not obtained, the decrease in leptin (by 22%) was not significant. As the body weight of the animals did not change, the decrease in leptin levels in the present study was independent of body weight. Although the animals in our study did not have obesity, hyperleptinaemia, or systemic leptin resistance, we believe that the main mechanism of the cardioprotective effect of a reduced blood leptin level is increased sensitivity of the myocardium and vascular endothelium to leptin. It cannot be ruled out that a certain contribution is made by a redistribution between the signalling pathways of leptin and insulin, which have a number of common components. In this regard, it should be noted that insulin and leptin can both stimulate the insulin receptor substrate (IRS)/phosphatidylinositol 3-kinase (PI3K)/AKT pathway, which is involved in the control of the survival and proliferative activity of various cell types.

### Ghrelin

In alimentary obesity, ghrelin resistance is associated with hyperleptinaemia, whereas decreased body weight and leptin levels can restore ghrelin receptor sensitivity^36^. The plasma ghrelin level after BS depends on the type of the anatomical modifications of the gastrointestinal tract and, possibly, the presence of postoperative vagus nerve dysfunction^37,38^. In obese rats, it has been shown that SG leads to a significant decrease in ghrelin levels^39^. In patients with T2DM, RYGB is more effective than SG and IT in reducing the acylated ghrelin levels^40^. However, Chinese investigators have shown that SG is more effective than RYGB in reducing ghrelin levels in obese patients^37^. It can be assumed that a decrease in the plasma levels of ghrelin and leptin after BS increases the sensitivity of ghrelin receptors. In the present study, the fasting ghrelin level was significantly reduced in the SG group compared with the CON and SHAM groups, whereas it was not significantly changed in the RYGB group (p = 0.08 and p = 0.06, respectively). In the RYGB and SG groups at 60 and 120 min of the OGTT, respectively, the decrease in the ghrelin level caused by glucose loading was more pronounced than that in the SHAM group. We showed that the overall course of the curves of plasma ghrelin levels in the SG and RYGB groups was lower than that in the control and SHAM groups. In the IT group, at 60 min of the OGTT, the changes in the plasma ghrelin level were smaller relative to the fasting ghrelin level, which indicates a slower ghrelin secretion in response to glucose stimulation. The cardioprotective properties of ghrelin have been proven in experimental and clinical studies^41–44^. The administration of ghrelin during reperfusion improved myocardial contractility and reduced the IS of isolated rat hearts^14^. Yang et al. administered ghrelin to rats in the early postinfarction period, which attenuated left ventricular remodelling and reduced the symptoms and severity of heart failure^45^. The mechanisms of the cardioprotective action of ghrelin are mediated by the activation of the IRS/PI3K/AKT and AMP-activated protein kinase pathways^46,47^ and a decrease in apoptosis^48^. In our study, it is likely that the decrease in the plasma ghrelin level in SG and RYGB rats caused an increase in the sensitivity of cardiac and vascular cells to ghrelin, which contributed to increased myocardial tolerance to ischaemia–reperfusion. Potential mechanisms of increased cell sensitivity to ghrelin include increased expression of ghrelin receptors (growth hormone secretagogue receptor 1a, GHSR1a), as well as suppression of ghrelin-induced receptor internalization and down-regulation. Desensitization of GHSR1a is usually caused by prolonged exposure to supraphysiological ghrelin concentrations, as shown in cell cultures expressing ghrelin receptor (Camiña et al., 2003, 2004), and, as a consequence, the sensitivity is restored at low concentrations of ghrelin^49,50^.

### GLP-1

GLP-1 is by far the most studied hormone in terms of cardiotropic effects in relation to the introduction of GLP-1 agonists and dipeptidyl peptidase-4 inhibitors into clinical practice^13,14,51^. GLP-1 enhances glucose-dependent insulin secretion, suppresses glucagon secretion, and exerts several extra-pancreatic effects owing to the presence of GLP-1 receptors in different cells, including endothelial and vascular smooth muscle cells^52^, cardiomyocytes, and endocardial cells^52^. Recently, Siraj et al. showed that the cardioprotective effect of GLP-1 may be due not to the direct interaction of this incretin with its membrane receptors but to the activation of the soluble form of adenylate cyclase in the smooth muscle and endothelial cells of coronary arteries, caused by the peptide GLP-1(28–36), a GLP-1 metabolite. This peptide is capable of penetrating vascular cells via macropinocytosis and interacts with mitochondrial trifunctional protein-α, through which it modulates the activity of soluble adenylate cyclase and cAMP-regulated effector proteins^53^.

Several experimental and clinical studies have shown the ability of GLP-1 to reduce the severity of ischaemia–reperfusion injury^54,55^, which is achieved through the activation of signalling pathways of ischaemic preconditioning^56^. It has been previously reported that GLP-1 can attenuate (reperfusion-induced) oxidative stress and activate the RISK (reperfusion injury survival kinase) and SAFE (survivor-activating factor enhancement) signalling cascades^55^, exerting a powerful cardioprotective effect through the inhibition of apoptosis and attenuation of cardiomyocyte necrosis^57^. In our study, an increase in the fasting GLP-1 levels in the RYGB and SG groups was observed compared with the CON group. Hence, it can be assumed that RYGB and SG may have long-term effects on the myocardium in the form of preconditioning, as well as can change the entire profile of gastrointestinal hormones.

Ischaemic preconditioning has previously been shown to reduce the magnitude of the no-reflow phenomenon^58^. We hypothesized that along with the infarct-limiting effect, increased GLP-1 secretion in animals after RYGB and SG can decrease the severity of the no-reflow phenomenon. A proof-of-concept study supporting our hypothesis showed the ability of the GLP-1 analogue liraglutide to reduce the no-reflow during percutaneous coronary intervention in patients with ST-elevation MI^59^. Our study demonstrated for the first time that RYGB and SG reduced the severity of no-reflow. The protective effect of BS is most likely caused by the vasoprotective effect of GLP-1, which has oxidative stress-reducing, anti-inflammatory^60^ and direct dose-dependent vasodilating effects^61^.

On the basis of literature data, we hypothesized that IT would also lead to a pronounced increase in GLP-1 secretion^62,63^. However, we observed that IT did not significantly affect the baseline GLP-1 level, and the operation itself was not effective in protecting against myocardial damage due to ischaemia and reperfusion. One of the possible reasons for this result is that we selected a model of normal Wistar rats (specific pathogen-free [SPF] rats) without obesity and diabetes mellitus^63^.

### Insulin

Several recent clinical and experimental studies have demonstrated the important role of insulin in maintaining myocardial resistance to ischaemia–reperfusion, especially in the presence of diabetes mellitus or insulin resistance^64^. Improvement of coronary perfusion, which protects cardiomyocytes from damage, has been reported as a potential cardioprotective mechanism of insulin through vasodilation and antiplatelet effects^65^. The anti-inflammatory effect is realized by suppressing tumour necrosis factor-α (TNF-α) production^66^ and is mediated through the activation of AKT protein kinase, which leads to a reduction in the size of the infarction and a decrease in myocardial contractile dysfunction. The suppression of inflammatory and oxidative stress–induced apoptosis upon the administration of insulin before ischaemia–reperfusion has been shown in in vitro and in vivo studies in the hearts of rabbits^67^ and dogs^68^. After the administration of insulin, a decrease in the size of necrosis was observed, along with an inhibition of apoptosis through the suppression of the activity of the pro-apoptotic enzyme caspase-3. Insulin administration caused a decrease in reperfusion injury through the activation of the protein kinase cascade, including PI3K, AKT, and p70-S6 kinase^69^. In 2011, Yu et al. showed that insulin has a cardioprotective effect through the activation of IRS/PI3K/AKT/endothelial nitric oxide synthase signalling and increased nitric oxide production^70^. On the basis of data showing that the cardioprotective effect of insulin in isolated rat hearts was blocked by rapamycin, an inhibitor of mammalian target of rapamycin (mTOR) protein kinase, it was concluded that mTOR is involved in the protective effects of the hormone^71^.

After BS, amelioration or normalization of hyperglycaemia, which is caused by increased incretin production, occurs long before the achievement of a clinically significant weight loss^72^. First, a few days after BS, an increase in insulin sensitivity in hepatocytes occurs against the background of nutrient intake deficiency. Peripheral insulin sensitivity increases later, together with a clinically significant weight loss, at 2–3 months after surgery, depending on the type of BS^73^.

In animal models of congenital T2DM (Goto–Kakizaki rats) without obesity, SG and IT resulted in decreased body weight and glucose levels, as well as increased ghrelin and GLP-1 levels and insulin sensitivity^62^. When IT was performed in animals without obesity and diabetes mellitus, decreased food intake and body weight, as well as increased insulin sensitivity were observed^63^. Our data did not show significant changes in fasting insulin levels, although an increase was observed in the SG group 60 min after glucose loading in the OGTT. In contrast, in the IT group, decreased insulin release was observed compared with that in the CON and SHAM groups.

This study had several methodological limitations. First, the animals were not fasted prior to induction of myocardial ischemia. Taking into account significantly higher values of blood glucose in SG and RYGB groups vs. SHAM group at 30 min of OGTT, one cannot exclude the possibility that perturbations in both blood glucose and insulin concentration might have been involved in cardioprotective effect of SG and RYGB. Therefore, it would be important to consider the addition of groups with fasting before infarction. However, these groups were not included in the present study. Second, we assessed changes in GLP-1, ghrelin, insulin, and leptin levels but did not analyze the levels of other humoral factors with putative cardioprotective and vasoprotective properties in the postoperative period, such as obestatin, adiponectin, and resistin. According to the literature, obestatin and adipokines (e.g., adiponectin and resistin) may exert cardioprotective effects in preclinical models of MI, and their levels are influenced by BS^74,75^. BS also improves responsiveness to endogenous thyroid hormones and decreases the levels of thyroid-stimulating hormone in obese patients^76,77^. These observations are important in the context of BS-mediated cardiac protection since it is known that thyroid hormones may increase myocardial tolerance to ischemia–reperfusion^78,79^.

Third, the mechanisms of the cardioprotective effect of BS were not investigated using pharmacological tools, such as the GLP-1 receptor antagonist exendin-α (9–39), in this study.

## Materials and methods

### Animals

Male SPF Wistar rats (Pushchino, Moscow Region, Russian Federation; weight, 260–300 g) were used throughout the study. The animals were maintained under a 12/12-h light/dark cycle with free access to food and drinking water, unless otherwise specified.

### Ethics

All procedures in this study were performed in accordance with the Guide for the Care and Use of Laboratory Animals (National Institutes of Health [NIH] Publication No. 85–23, revised 2011) and the European Convention for the Protection of Vertebrate Animals Used for Experimental and Other Scientific Purposes. The Institutional Animal Care and Use Committee at Almazov National Medical Research Centre approved the study protocol (protocol no. 19–19; 25 November 2019). All efforts were made to protect the animals and minimize their suffering during the study. The experiments complied with the ARRIVE guidelines (http://www.nc3rs.org/ARRIVE).

### Reagents

All chemicals used were of analytical grade and were purchased from Sigma-Aldrich (St. Louis, MO, USA), unless otherwise specified.

### Experimental design

**Figure 7:**
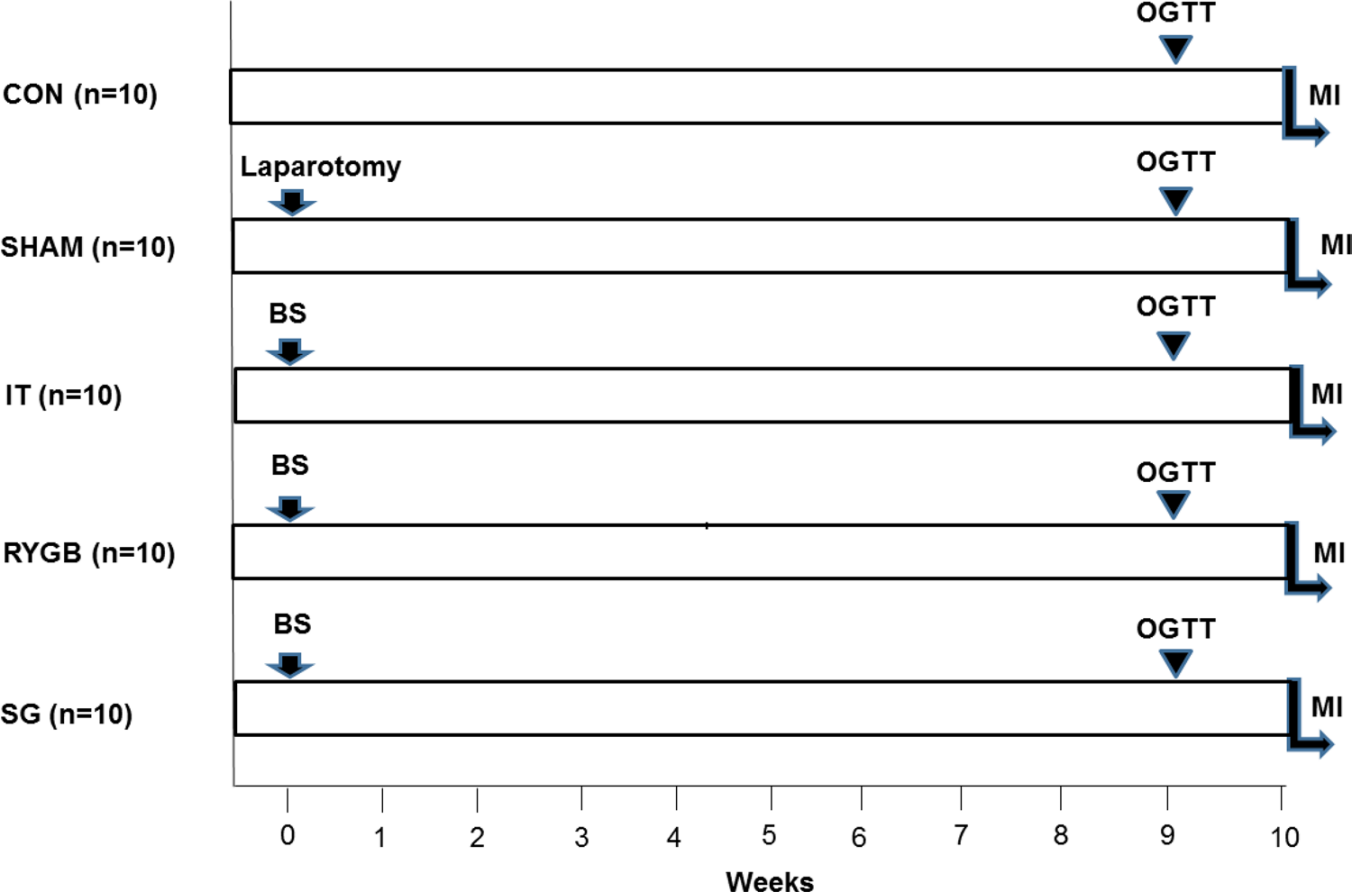
Experimental design. *BS* bariatric surgery, *MI* myocardial infarction, *OGTT* oral glucose tolerance test, *CON* control, *SHAM* sham surgery, *IT* ileal transposition, *RYGB* Roux-en-Y gastric bypass, *SG* sleeve gastrectomy.

The animals were randomly divided into five groups (Fig. 7): CON (n = 10), SHAM (n = 10), IT (n = 10), RYGB (n = 10), and SG (n = 10). After surgery, the animals were fed regularly and allowed to recover for 10 weeks before MI induction. At 9 weeks postoperatively, an OGTT was performed together with measurements of serum GLP-1, ghrelin, leptin, and insulin levels (Fig. 7). The weight of the animals was determined each week after surgery. Food consumption was assessed as the mean of the measurements made for 3 consecutive days at 8 weeks after surgery.

### BS procedures

The animals were deprived of food for 12 h before surgery, but allowed free access to water. All surgical interventions were performed under isoflurane anaesthesia using an aseptic technique. The detailed technique of BS in rats has been described elsewhere^80,81^. In SG, a midline laparotomy was performed and approximately 70% of the stomach was removed, including most of the fundal portion. RYGB included surgical exclusion of the major part of the stomach and the proximal small intestine. In IT, a 10-cm ileal segment was translocated to the proximal jejunum. The stomach and intestines were sutured using a polypropylene (Prolene 6–0; Ethicon, USA) hand-sewn suture. In sham-operated animals, only midline laparotomy was performed. The abdominal wound was closed in layers using a continuous atraumatic suture (Prolene 6–0, Ethicon). Immediately after wound closure, the animals were rehydrated through the subcutaneous administration of 8–10 mL sterile 0.9% saline and placed in a thermostatic recovery chamber (37 °C). Upon resuming oral nutrition, all animals were fed a liquid diet for 3 days and a standard diet thereafter.

### OGTT

The rats were fasted for 12 h, after which a bolus of glucose (2 g/kg) was administered. The blood glucose levels were measured in samples obtained from the tail vein before (0 min) and 30, 60, 90, and 120 min after glucose loading, using a glucometer (OneTouch Verio Pro; Johnson & Johnson, Switzerland) and test strips (One Touch Verio; USA). Trapezoidal integration was used to calculate the AUCs in the OGTT.

### Assessment of hormonal parameters and homeostasis of insulin resistance

Blood GLP-1 levels were measured in the samples obtained from the tail vein before (0 min) and 15, 30, 60, and 120 min after glucose loading. Blood ghrelin, leptin, and insulin levels were measured in the samples obtained from the tail vein before (0 min) and 60 and 120 min after glucose loading. The plasma levels of insulin, leptin, GLP-1, and ghrelin were measured using the Rat Insulin ELISA kit (Mercodia AB, Sweden), ELISA for Leptin (Rat), ELISA kit for GLP-1, and ELISA kit for Ghrelin (Rat) (Cloud-Clone Corp., USA), respectively. Trapezoidal integration was used to calculate the AUC of GLP-1 levels. The homeostasis of insulin resistance index ([fasting glucose × fasting insulin] / 22.5) was calculated to estimate insulin resistance^82^. In this study, we evaluated insulin secretion in response to glucose load over a very limited period of time (≤ 120 min) in order to study the profile of insulin release and correlate insulin concentration with the blood levels of glucose and other hormones. Samples were immediately frozen at -80 °C or immediately used for analysis, which prevented insulin degradation. Therefore, proinsulin C-peptide levels were not measured.

### Myocardial ischaemia–reperfusion model

This part of the study was performed in accordance to IMPACT guidelines^83^ and guidelines for rigor and reproducibility in preclinical and clinical studies on cardioprotection^84^. The animals were allowed free access to water and food. General anaesthesia was induced through the inhalation of 5% isoflurane using a low-flow gas apparatus (SomnoSuite; Kent Scientific, Torrington, CT, USA). After the induction of anaesthesia, the animals were tracheotomised and ventilated (SAR-830P; CWE Inc., Ardmore, PA, USA) using gas containing 2–3% isoflurane, 35% oxygen, and 62–63% room air, with a tidal volume of 2 mL/100 g and a rate of approximately 60 breaths/min. Body temperature was maintained at 37.0 ± 0.5 °C using a feedback-controlled heating pad (TCAT-2LV; Physitemp Instruments Inc., Clifton, NJ, USA). The left carotid artery and right femoral vein were cannulated for mean arterial pressure (MAP) measurement and drug injection, respectively. Lead II of the electrocardiogram was monitored to record heart rate (HR) and arrhythmias. A 6–0 polypropylene thread was placed around the left coronary artery via a left anterior thoracotomy, and the ends were passed through a polyethylene tube as an occluder. After surgical preparation and a 30-min stabilisation period, the animals were subjected to 30 min of coronary artery occlusion followed by 120 min of reperfusion. The exclusion criteria were MAP < 50 mmHg or HR < 300 at any time point during the experiment. MAP and HR were measured at baseline; 15 min of ischaemia; and 40, 90, and 120 min of reperfusion. Thereafter, the animals were euthanized and the IS, no-reflow area, and blood stasis zones were assessed.

### IS measurement

The left coronary artery was re-occluded, followed by the administration 0.5 mL of 5% Evans Blue through the femoral vein to identify the AR. The hearts were excised and cut into 2-mm-thick slices parallel to the atrioventricular groove. The basal surface of each slice was digitally photographed. The slices were immersed in a 1% solution of 2,3,5-triphenyltetrazolium chloride at 37 °C (pH 7.4) for 15 min and photographed again to identify the IS. The images were digitized using ImageJ 1.34 s software (NIH, Bethesda, MD, USA). The AR was expressed as a percentage of the whole slice, and the IS was expressed as a percentage of the AR. The sizes of the AR and IS for each heart were obtained by summing the measurements of the slices and calculating the mean values. Animals with an AR of < 15% were excluded from the study. IS measurements and data analyses were performed by an investigator blinded to the study groups. The animals excluded from the analysis of IS were also excluded from no-reflow analysis. Biochemical parameters of the blood were analyzed in all animals included at the moment of OGTT in order to increase the statistical power.

### Assessment of no-reflow and blood stasis areas

The animals were injected with 1 mL of 1 mg/kg indocyanine green (Pulsion Medical Systems, AG, Germany) at 90 min of reperfusion. Infusion of indocyanine green did not cause any effects on the hemodynamics. The near-infrared fluorescence of the 2-mm-thick myocardial slices was registered using an optical system providing an excitation wavelength of 780–810 nm and registration in the range of 820–900 nm, as previously described^85^. Image analysis was performed (RSScam–Neuro 1.4.623) after image acquisition with IVIS Lumina Series III (PerkinElmer, CA, USA), allowing the quantification of fluorescence intensity. No-reflow area were defined as defects in the accumulation of indocyanine green within the area of necrosis and were expressed as a percentage of the AR. To assess the severity of blood stasis, we performed Mallory staining (BioVitrum, St-Petersburg, Russian Federation) of the heart slices. The slides were analysed using an optical upright microscope (Nikon Eclipse Ni-U; Nikon Corporation, Tokyo, Japan) at 200 × magnification, and morphometry was performed with NIS-Elements BR 4.3 (Nikon Corporation, Tokyo, Japan). The blood stasis area was expressed as a percentage of the total slice area after quantifying 10 visual fields within the no-reflow area. The slides were analysed by a pathologist who was blinded to the group assignments.

### Statistical analysis

All data are presented as mean ± standard deviation. Hemodynamic data are expressed as median (25th quartile, 75th quartile). Statistical analysis was performed using SPSS (version 12.0; IBM Corporation, Armonk, NY, USA). Differences in continuous data were tested using repeated-measures analysis of variance, followed by a Tukey post-hoc test. The Kruskal–Wallis test was used to determine the overall differences between groups in IS, no-reflow area, areas of blood stasis, and AUCs. Further, pairwise comparisons between groups were performed using the non-parametric Mann–Whitney U test. Statistical significance was set at p < 0.05.

## Conclusions

In this study, we demonstrated that both RYGB and SG lead to an increase in myocardial tolerance to ischemia–reperfusion injury in non-obese, non-diabetic rats as evidenced from smaller IS and no-reflow area. The infarct-limiting effect of RYGB and SG was associated with increased fasting GLP-1 levels and decreased fasting levels of leptin and ghrelin. In contrast, IT failed to protect the heart from infarction but still decreased the severity of no-reflow. It might be assumed that the observed cardioprotective effect of RYGB and SG is mediated by increased GLP-1 level as well as improved sensitivity of cardiac cells to leptin, insulin, and ghrelin. The paradigm shift in BS from being considered a purely weight loss surgery to being a type of metabolic surgery is an additional rationale for the use of BS to reduce mortality from cardiovascular events.

## Data Availability

All data generated or analyzed during this study are included in this published article (and its Supplementary Information files).

## References

[1] Buchwald H (2004). Bariatric surgery: A systematic review and meta-analysis. JAMA.

[2] Kokkinos A, Tsilingiris D, le Roux CW, Rubino F, Mantzoros CS (2019). Will medications that mimic gut hormones or target their receptors eventually replace bariatric surgery?. Metabolism.

[3] Sjöström L (2013). Review of the key results from the Swedish Obese Subjects (SOS) trial: A prospective controlled intervention study of bariatric surgery. J. Intern. Med..

[4] Sheng B (2017). The long-term effects of bariatric surgery on type 2 diabetes remission, microvascular and macrovascular complications, and mortality: A systematic review and meta-analysis. Obes. Surg..

[5] Kuno T, Tanimoto E, Morita S, Shimada YJ (2019). Effects of bariatric surgery on cardiovascular disease: A concise update of recent advances. Front. Cardiovasc. Med..

[6] Aminian A (2019). Bariatric surgery is associated with a lower rate of death after myocardial infarction and stroke: A nationwide study. Diabetes Obes. Metab..

[7] Buchwald H, Varco RL (1978). Metabolic Surgery.

[8] Bohdjalian A (2006). One-year experience with Tantalus^TM^: A new surgical approach to treat morbid obesity. Obes. Surg..

[9] Berthoud H-R, Shin AC, Zheng H (2011). Obesity surgery and gut-brain communication. Physiol. Behav..

[10] Hutch CR, Sandoval DA (2017). Physiological and molecular responses to bariatric surgery: Markers or mechanisms underlying T2DM resolution?. Ann. N. Y. Acad. Sci..

[11] Farkhondeh T (2020). An overview of the role of adipokines in cardiometabolic diseases. Molecules.

[12] Osto E (2015). Rapid and body weight-independent improvement of endothelial and high-density lipoprotein function after Roux-en-Y gastric bypass. Circulation.

[13] Widiarti W, Sukmajaya AC, Nugraha D, Alkaff FF (2021). Cardioprotective properties of glucagon-like peptide-1 receptor agonists in type 2 diabetes mellitus patients: A systematic review. Diabetes Metab. Syndr..

[14] Zafeiropoulos S (2021). Cardio-protective effects of glucagon-like peptide-1 receptor agonists: An overview of systematic reviews and publication overlap. Curr. Pharm. Des..

[15] Korniushyn OV (2016). Pathophysiological aspects of pleiotropic effects of gastrointestinal hormones. Eksperimental’naia i Klinicheskaia Gastroenterologiia = Exp. Clin. Gastroenterol..

[16] Sawashita Y (2020). Remote ischemic preconditioning reduces myocardial ischemia–reperfusion injury through unacylated ghrelin-induced activation of the JAK/STAT pathway. Basic Res. Cardiol..

[17] Yuan M-J, Li W, Zhong P (2021). Research progress of ghrelin on cardiovascular disease. Biosci. Rep..

[18] Sala PC, Torrinhas RS, Giannella-Neto D, Waitzberg DL (2014). Relationship between gut hormones and glucose homeostasis after bariatric surgery. Diabetol. Metab. Syndr..

[19] Kornyushin OV (2021). Effects of three types of bariatric interventions on myocardial infarct size and vascular function in rats with type 2 diabetes mellitus. Life Sci..

[20] Kain D (2018). Cardiac leptin overexpression in the context of acute MI and reperfusion potentiates myocardial remodeling and left ventricular dysfunction. PLoS ONE.

[21] Galagudza MM, Nekrasova MK, Syrenskii AV, Nifontov EM (2007). Resistance of the myocardium to ischemia and the efficacy of ischemic preconditioning in experimental diabetes mellitus. Neurosci. Behav. Physiol..

[22] Kristiansen SB (2019). Impact of hyperglycemia on myocardial ischemia–reperfusion susceptibility and ischemic preconditioning in hearts from rats with type 2 diabetes. Cardiovasc. Diabetol..

[23] Kornyushin OV, Galagudza MM, Neymark AE, Grineva AY, Babenko EN (2015). Ileal transposition in surgical treatment for type 2 diabetes mellitus. Diabetes Mellitus.

[24] Ashrafian H, le Roux CW, Darzi A, Athanasiou T (2008). Effects of bariatric surgery on cardiovascular function. Circulation.

[25] Edwards C, Hindle AK, Fu S, Brody F (2011). Downregulation of leptin and resistin expression in blood following bariatric surgery. Surg. Endosc..

[26] Abd Alkhaleq H (2020). Leptin modulates gene expression in the heart and cardiomyocytes towards mitigating ischemia-induced damage. Exp. Cell Res..

[27] Moro C (2011). Inhibition of cardiac leptin expression after infarction reduces subsequent dysfunction. J. Cell Mol. Med..

[28] Smith CCT (2010). Leptin-induced cardioprotection involves JAK/STAT signaling that may be linked to the mitochondrial permeability transition pore. Am. J. Physiol..

[29] Dixon RA, Davidson SM, Wynne AM, Yellon DM, Smith CCT (2009). The cardioprotective actions of leptin are lost in the Zucker obese (fa/fa) rat. J. Cardiovasc. Pharmacol..

[30] Heusch G (2006). Obesity: A risk factor or a RISK factor for myocardial infarction?. Br. J. Pharmacol..

[31] Xu T, Liu S, Wang X (2010). Amelioration of myocardial ischemia/reperfusion injury by leptin pretreatment and ischemic preconditioning in mouse. Zhongguo wei zhong bing ji jiu yi xue = Chinese critical care medicine Zhongguo weizhongbing jijiuyixue.

[32] Purdham DM (2008). A neutralizing leptin receptor antibody mitigates hypertrophy and hemodynamic dysfunction in the postinfarcted rat heart. Am. J. Physiol..

[33] Engin A, Engin AB, Engin A (2017). Diet-Induced Obesity and the Mechanism of Leptin Resistance BT: Obesity and Lipotoxicity.

[34] Zhang R (2016). Effects of cereal fiber on leptin resistance and sensitivity in C57BL/6J mice fed a high-fat/cholesterol diet. Food Nutr. Res..

[35] Nausheen S, Shah IH, Pezeshki A, Sigalet DL, Chelikani PK (2013). Effects of sleeve gastrectomy and ileal transposition, alone and in combination, on food intake, body weight, gut hormones, and glucose metabolism in rats. Am. J. Physiol..

[36] Zigman JM, Bouret SG, Andrews ZB (2016). Obesity impairs the action of the neuroendocrine ghrelin system. Trends Endocrinol. Metab..

[37] Yang J (2018). Effect of laparoscopic Roux-en-Y gastric bypass versus laparoscopic sleeve gastrectomy on fasting gastrointestinal and pancreatic peptide hormones: A prospective nonrandomized trial. Surg. Obes. Relat. Dis..

[38] Wang Y, Chen J, Wu X-T (2020). No effect on change in fasting ghrelin at ≤ 12 months and increased at ≥ 24 months after Roux-en-Y gastric bypass. Obes. Surg..

[39] Moncada R (2016). Sleeve gastrectomy decreases body weight, whole-body adiposity, and blood pressure even in aged diet-induced obese rats. Obes. Surg..

[40] Malin SK (2014). Improved acylated ghrelin suppression at 2 years in obese patients with type 2 diabetes: Effects of bariatric surgery vs standard medical therapy. Int. J. Obes..

[41] Mao Y (2012). Ghrelin prevents incidence of malignant arrhythmia after acute myocardial infarction through vagal afferent nerves. Endocrinology.

[42] Soeki T (2013). Ghrelin protects the heart against ischemia-induced arrhythmias by preserving connexin-43 protein. Heart Vessels.

[43] Sullivan R (2021). Regional differences in the ghrelin-growth hormone secretagogue receptor signalling system in human heart disease. CJC Open.

[44] Sun N (2021). Ghrelin attenuates depressive-like behavior, heart failure, and neuroinflammation in postmyocardial infarction rat model. Eur. J. Pharmacol..

[45] Yang C, Liu Z, Liu K, Yang P (2014). Mechanisms of ghrelin anti-heart failure: Inhibition of Ang II-induced cardiomyocyte apoptosis by down-regulating AT1R expression. PLoS ONE.

[46] Chen Y (2019). Pretreatment of ghrelin protects H9c2 cells against hypoxia/reoxygenation-induced cell death via PI3K/AKT and AMPK pathways. Artif. Cells Nanomed. Biotechnol..

[47] Lu W (2021). Ghrelin inhibited pressure overload-induced cardiac hypertrophy by promoting autophagy via CaMKK/AMPK signaling pathway. Peptides.

[48] Iglesias MJ (2004). Growth hormone releasing peptide (ghrelin) is synthesized and secreted by cardiomyocytes. Cardiovasc. Res..

[49] Camiña JP (2003). Regulation of ghrelin secretion and action. Endocrine.

[50] Camiña JP (2004). Desensitization and endocytosis mechanisms of ghrelin-activated growth hormone secretagogue receptor 1a. Endocrinology.

[51] Bradic J (2021). Dipeptidyl peptidase 4 inhibitors attenuate cardiac ischaemia–reperfusion injury in rats with diabetes mellitus type 2. Clin. Exp. Pharmacol. Physiol..

[52] Ahrén B (2004). GLP-1 and extra-islet effects. Hormon. Metab. Res. = Hormon- Stoffwechselforschung = Hormones et Metabolisme.

[53] Siraj MA (2020). Cardioprotective GLP-1 metabolite prevents ischemic cardiac injury by inhibiting mitochondrial trifunctional protein-α. J. Clin. Investig..

[54] Bose AK, Mocanu MM, Carr RD, Brand CL, Yellon DM (2005). Glucagon-like peptide 1 can directly protect the heart against ischemia/reperfusion injury. Diabetes.

[55] Giblett JP, Clarke SJ, Dutka DP, Hoole SP (2016). Glucagon-like peptide-1: A promising agent for cardioprotection during myocardial ischemia. JACC.

[56] Hausenloy DJ, Yellon DM (2004). New directions for protecting the heart against ischaemia-reperfusion injury: Targeting the Reperfusion Injury Salvage Kinase (RISK)-pathway. Cardiovasc. Res..

[57] Skyschally A, Schulz R, Heusch G (2008). Pathophysiology of myocardial infarction: Protection by ischemic pre- and postconditioning. Herz.

[58] Chen WR (2016). Effects of liraglutide on reperfusion injury in patients with ST-segment–elevation myocardial infarction. Circulation.

[59] Hattori Y (2010). RETRACTED ARTICLE: A glucagon-like peptide-1 (GLP-1) analogue, liraglutide, upregulates nitric oxide production and exerts anti-inflammatory action in endothelial cells. Diabetologia.

[60] Heusch G (2011). Obesity and inflammatory vasculopathy. Arterioscler. Thromb. Vasc. Biol..

[61] Sabench Pereferrer F (2009). The effects of ileal transposition, gastrojejunal bypass and vertical gastroplasty on the regulation of ingestion in an experimental obesity model associated with diabetes mellitus type 2. Cir. Esp..

[62] Wang H (2019). Therapeutic effects of sleeve gastrectomy and ileal transposition on type 2 diabetes in a non-obese rat model by regulating blood glucose and reducing ghrelin levels. Med. Sci. Monit..

[63] Oh TJ, Lee H-J, Cho YM (2016). Ileal transposition decreases plasma lipopolysaccharide levels in association with increased L cell secretion in non-obese non-diabetic rats. Obes. Surg..

[64] Bianchi VE (2020). Caloric restriction in heart failure: A systematic review. Clin. Nutr. ESPEN.

[65] Masoumi G (2014). Effects of moderate glycemic control in type II diabetes with insulin on arterial blood gas parameters following coronary artery bypass graft surgery. Res. Cardiovasc. Med..

[66] Fu F (2015). Direct evidence that myocardial insulin resistance following myocardial ischemia contributes to post-ischemic heart failure. Sci. Rep..

[67] Zhang HF (2004). Role of insulin in the anti-apoptotic effect of glucose-insulin-potassium in rabbits with acute myocardial ischemia and reperfusion. Apoptosis.

[68] Yu Q (2014). Effective glycaemic control critically determines insulin cardioprotection against ischaemia/reperfusion injury in anaesthetized dogs. Cardiovasc. Res..

[69] Jonassen AK, Mjøs OD, Sack MN (2004). p70s6 kinase is a functional target of insulin activated Akt cell-survival signaling. Biochem. Biophys. Res. Commun..

[70] Yu Q, Gao F, Ma XL (2011). Insulin says NO to cardiovascular disease. Cardiovasc. Res..

[71] Fuglesteg BN, Tiron C, Jonassen AK, Mjøs OD, Ytrehus K (2009). Pretreatment with insulin before ischaemia reduces infarct size in Langendorff-perfused rat hearts. Acta Physiol. (Oxf.).

[72] Penna C (2017). Obestatin regulates cardiovascular function and promotes cardioprotection through the nitric oxide pathway. J. Cell Mol. Med..

[73] Alkofide H, Huggins GS, Ruthazer R, Beshansky JR, Selker HP (2015). Serum adiponectin levels in patients with acute coronary syndromes: Serial changes and relation to infarct size. Diabetes Vasc. Dis. Res..

[74] Drucker DJ (2018). Mechanisms of action and therapeutic application of glucagon-like peptide-1. Cell Metab..

[75] Jacobsen SH (2012). Changes in gastrointestinal hormone responses, insulin sensitivity, and beta-cell function within 2 weeks after gastric bypass in non-diabetic subjects. Obes. Surg..

[76] Juiz-Valiña P (2020). Central resistance to thyroid hormones in morbidly obese subjects is reversed after bariatric surgery-induced weight loss. J. Clin. Med..

[77] Neves JS (2018). Effect of weight loss after bariatric surgery on thyroid-stimulating hormone levels in patients with morbid obesity and normal thyroid function. Obes. Surg..

[78] Lieder HR (2021). Cardioprotection by post-conditioning with exogenous triiodothyronine in isolated perfused rat hearts and isolated adult rat cardiomyocytes. Basic Res. Cardiol..

[79] Louzada RA (2021). 3,5-Diiodothyronine protects against cardiac ischaemia–reperfusion injury in male rats. Exp. Physiol..

[80] Sabench Pereferrer F, Hernàndez Gonzàlez M, Del Castillo Déjardin D (2011). Experimental metabolic surgery: Justification and technical aspects. Obes. Surg..

[81] Bueter M, Abegg K, Seyfried F, Lutz TA, le Roux CW (2012). Roux-en-Y gastric bypass operation in rats. JoVE.

[82] Dolo PR (2019). The effect of gastric bypass with a distal gastric pouch on glucose tolerance and diabetes remission in type 2 diabetes Sprague-Dawley rat model. Obes. Surg..

[83] Lecour S (2021). IMproving preclinical assessment of cardioprotective therapies (IMPACT) criteria: Guidelines of the EU-CARDIOPROTECTION COST Action. Basic Res. Cardiol..

[84] Bøtker HE, Hausenloy D, Andreadou I (2018). Practical guidelines for rigor and reproducibility in preclinical and clinical studies on cardioprotection. Basic Res. Cardiol..

[85] Sonin D (2017). In vivo visalization and ex vivo quantification of experimental myocardial infarction by indocyanine green fluorescence imaging. Biomed. Opt. Express.

